# Medial Clavicle Physeal Fracture in a 15-Year-Old Male: A Case Report

**DOI:** 10.5811/cpcem.50643

**Published:** 2026-01-14

**Authors:** Rochelle Kofman, Ryan Allen, Addison B. Smartt, Kevin M. Drechsel, Jordan R. Pollock, Douglas Rappaport

**Affiliations:** * Mayo Clinic Alix School of Medicine, Phoenix, Arizona; † Mayo Clinic Arizona, Department of Emergency Medicine, Phoenix, Arizona; ‡ Mayo Clinic Arizona, Department of Radiology, Phoenix, Arizona

**Keywords:** sternoclavicular joint, pediatric sports injury, physeal fracture, case report

## Abstract

**Introduction:**

Sternoclavicular joint injuries are rare and potentially life-threatening injuries due to their proximity to vital mediastinal structures. In adolescents, skeletal immaturity can add complexity to the injury due to potential involvement of the physis. A physeal fracture with displacement can appear as a dislocation on imaging, also known as pseudo-dislocation. Additionally, this anatomic area is difficult to visualize with plain radiographs, which can result in misdiagnosis and delayed treatment.

**Case Report:**

We present a case of a 15-year-old male athlete who presented to the emergency department with severe right clavicular pain four hours after sustaining a football injury. Plain radiographs obtained at an outside facility as well as repeat plain radiographs at our facility showed no evidence of fracture or dislocation. The patient’s degree of pain and physical exam findings prompted further imaging with computed tomography (CT), ultimately revealing a physeal fracture of the medial right clavicle with posterior and superior displacement.

**Conclusion:**

Sternoclavicular joint injuries in skeletally immature patients are complex and require immediate diagnosis and intervention. Plain radiographs are often unreliable in recognizing these injuries and, in our case, the physeal fracture with displacement was not radiographically apparent on two separate occasions. Advanced imaging with CT revealed the diagnosis, highlighting the importance of a detailed physical exam and for physicians to maintain a high index of clinical suspicion when evaluating adolescents with high-impact trauma, even in the setting of negative plain radiographs.

## INTRODUCTION

Sternoclavicular joint (SCJ) injuries are relatively uncommon, constituting less than 1% of all clavicular injuries.^1^ In adolescents as many as 75% of SCJ injuries are posterior, which can be potentially serious or even life-threatening due to damage to vital underlying structures including the trachea, esophagus, and great vessels.^1^, ^2^ Computed tomography (CT) is the imaging modality of choice for injuries of this nature, given poor visualization of mediastinal structures with plain radiographs, as well as poor sensitivity of plain radiographs for detecting SCJ fracture or dislocation.^3^–^5^ While CT and magnetic resonance imaging (MRI) have higher diagnostic accuracy and sensitivity evaluating for SCJ injury, clinicians may not be aware of the need for advanced imaging in the setting of a negative plain radiographs for this injury.^6^–^8^

In pediatric populations still undergoing skeletal development, the incidence and categorization of this type of injury are often unclear. Typically, these injuries, described as either SCJ dislocation or medial clavicular fracture, occur during contact sports.^8^, ^9^ Some studies suggest that fractures are far more common than dislocation in the setting of posterior SCJ injury, although one retrospective review of 48 adolescent patients suggests the two have prevalence.^8^ Cases of SCJ dislocation with accompanying fracture are exceedingly rare, although some are described in the literature and require advanced imaging such as CT or MRI to properly diagnose.^10^, ^11^

Here, we present a case of medial clavicle physeal fracture with posterior and superior displacement in a 15-year-old patient after a football injury requiring open reduction and internal fixation.

## CASE REPORT

A 15-year-old male presented to the emergency department (ED) with right clavicular pain approximately four hours following a football injury. The patient described the event as falling after jumping for a ball, with another player falling on top of him. He broke the fall with his right elbow, which resulted in immediate pain in the right clavicular area. Initial plain radiographs at an outside facility did not reveal any fractures. However, further care was pursued due to the severity of his pain.

Upon arrival to the ED, vitals were within normal limits. The patient described the pain as 8/10 in severity and localized to the right clavicle, with significant tenderness and mild right anterior neck pain. On physical examination, there was tenderness and a palpable deformity over the right mid-to-proximal clavicle. A depressive deformity was noted at the SCJ. No crepitus or subcutaneous emphysema was noted. The right radial pulse was intact, and grip strength was preserved. There was no tenderness over the right acromioclavicular joint or anterior shoulder. No midline cervical spine tenderness or tracheal deviation was appreciated. Pulmonary exam showed normal breath sounds, and the patient was in no acute respiratory distress.

**Image 1A and 1B:**
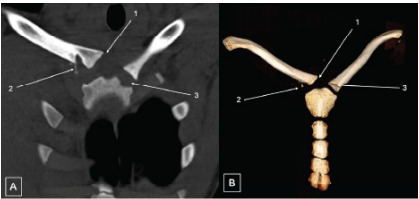
Superior displacement of the right medial clavicle relative to the sternum shown by arrow one on coronal computed tomography (CT) without contrast (A) and three-dimensional CT reconstruction (B). Relative to the displaced clavicle, the epiphyseal ossification is in the expected anatomic location, suggesting fracture of the physis with displacement of the ossified clavicle, shown by arrow two. The unaffected left clavicle is anatomically aligned, shown by arrow three.

**Image 2:**
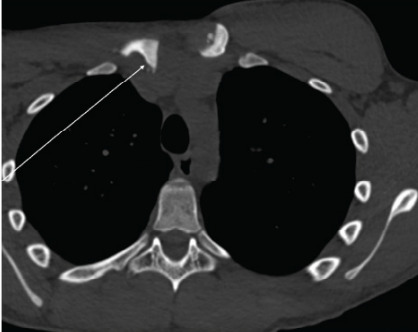
Axial computed tomography without contrast showing posterior displacement of the right medial clavicle relative to the left clavicle, noted by arrow.

Consistent with the radiographs obtained earlier, clavicle radiographs obtained in the ED again showed no sign of fracture or dislocation, with reports of normal acromioclavicular and coracoclavicular intervals and unremarkable appearance of acromioclavicular and glenohumeral joints. Further investigation with chest CT was pursued due to persistent pain and concern for deeper injury, which revealed a right SCJ dissociation with approximately 1.4 cm of posterior displacement of the medial clavicle, along with a possible small adjacent avulsion fragment suspicious for physeal fracture (Images 1–2). A small hematoma was noted anteroinferior to the right clavicular head, without evidence of pneumothorax, pulmonary contusion, mediastinal hematoma, or vascular injury.

*CPC-EM Capsule*

What do we already know about this clinical entity?

*Posterior sternoclavicular joint (SCJ) injuries are rare, often subtle on imaging, and can be life-threatening due to proximity to vital mediastinal structures.*

What makes this presentation of disease reportable?

*A physeal fracture with posterior SCJ displacement in an adolescent, which was missed on initial plain radiograph, was confirmed with computed tomography (CT) due to physician’s clinical suspicion.*

What is the major learning point?

*High clinical suspicion is critical; CT is the imaging modality of choice, even in the setting of negative plain radiographs in suspected SCJ trauma.*

How might this improve emergency medicine practice?

*Earlier CT use in the emergency department and orthopedic consult for at-risk mechanisms could improve outcomes in pediatric SCJ trauma cases.*

Orthopedic and thoracic surgery teams were consulted and ultimately recommended the patient be evaluated at a pediatric center. After discussion with the accepting tertiary-care pediatric facility, the patient was placed in a shoulder immobilizer and determined to be stable for transfer by private vehicle. Upon arrival to the pediatric ED, the evaluating pediatric orthopedic team proceeded with immediate surgical intervention, including open reduction and internal fixation of the SCJ injury. Physeal fracture with posterior superior displacement of the clavicle was confirmed intraoperatively. The operative course was uncomplicated, and he tolerated the procedure and anesthesia well. He was discharged the same day in a soft sling for immobilization, with plans to follow up and begin physical therapy six weeks postoperatively. A full recovery was anticipated after three months.

## DISCUSSION

Posterior sternoclavicular joint displacements are rare injuries that carry a significant clinical risk due to the proximity to vital mediastinal structures such as subclavian and brachiocephalic vessels, trachea, and esophagus. These are all at risk of compression or damage in this type of injury.^1^,^2^ Posterior SCJ injuries carry a higher risk for life-threatening complications such as vascular injury and airway injury.^12^,^13^ The critical nature of these complications makes prompt diagnosis of this injury essential and management by multidisciplinary care teams necessary to prevent potentially fatal complications.

Initial imaging with plain radiographs is often insufficient in identifying posterior SCJ injuries because of the joint’s deep anatomic location and overlapping of the clavicle with thoracic structures.^5^ A high index of clinical suspicion for SCJ injury should prompt further advanced imaging even in the setting of negative plain radiographs. Computed tomography is the gold standard imaging modality for SCJ injuries as it allows for clear delineation of joint alignment, physeal involvement, and clear views of mediastinal structures.^5^,^13^

Despite this, the diagnosis is sometimes still missed or delayed in the setting of negative plain radiographs, with missed diagnosis rates up to 23–25% in pediatric cohorts. This may be due to physicians’ lack of awareness of the limitation of plain radiographs for SCJ injuries and lack of standardized protocols for such injuries.^8^, ^14^ Retrospective reviews have shown that CT is often delayed until after orthopedic consultation, rather than being obtained at the initial presentation, which further contributes to diagnostic delays.^15^ The early use of CT in our patient confirmed the diagnosis, ensured the proper specialists were involved, and facilitated an effective and safe transfer of the patient to a tertiary-care pediatric center.

Skeletal immaturity added complexity to this patient’s injury and, in general, makes interpreting SCJ injuries in the pediatric population more challenging. The medial clavicular physis typically fuses between 22–25 years of age; therefore, it may be difficult to radiographically distinguish physeal fractures from dislocations in adolescents.^8^ In this patient, CT revealed a small bony fragment adjacent to the displaced clavicle, which suggested physeal involvement. Differentiating between physeal fractures and joint dislocations is crucial, as their treatment and management are vastly different, particularly in children.^9^,^10^ However, sometimes this distinction cannot be made even with advanced imaging, and the diagnosis must be confirmed intraoperatively. In this patient, surgery confirmed the presence of a physeal fracture with posterior displacement.

The mechanism that led to this patient’s injury is classic for a SCJ injury with displacement. Axial loading through a fall onto the elbow aligns with the literature that describes the most common etiologies of this injury.^9^,^14^ Contact sports such as football and wrestling are the leading sources of pediatric SCJ trauma.^6^,^8^ In young athletes presenting with clavicle pain following high-energy impacts, physicians should carefully examine the SCJ, even in the setting of negative initial plain radiographs and the absence of obvious vascular or airway compromise.

Management of suspected posterior SCJ displacements or physeal fractures with posterior displacement requires a multidisciplinary approach due to its complex nature and risk profile. Thoracic surgery should be consulted due to the risk of mediastinal injuries, as well as orthopedic surgery. Closed reduction is preferred within 48 hours of injury and should be performed with cardiothoracic or vascular surgery backup. Open reduction is indicated if closed reduction fails or is delayed. While non-operative management may be considered if alignment is preserved and there are no complications, most displaced injuries require surgical stabilization.^2^, ^9^, ^14^ In our case, consultation with a pediatric care team was needed due to the skeletally immature nature of this patient, and prompt transfer to a pediatric center was safely coordinated.

This case contributes to the growing literature on pediatric SCJ injuries. While similar cases of dislocation-fractures and pseudo-dislocations have been reported, this remains a rare injury and, importantly, highlights the significance of the emergency physician’s clinical suspicion to pursue further imaging, despite negative initial films. This case reinforces the key point that a negative initial plain radiograph does not rule out SCJ injury. Clinical suspicion and awareness of these injuries must prompt further investigation with more advanced imaging early in the care timeline to prevent delays in diagnosis and treatment.

## CONCLUSION

This was a rare case of a posterior sternoclavicular joint displacement with physeal involvement in an adolescent football player; a diagnosis originally missed on initial plain radiographs. Computed tomography is often underused in emergency settings for SCJ injuries, despite the known limitations of plain radiographs in identifying posterior displacement, and the diagnosis is often missed or delayed. This case reinforces the need for early advanced imaging, even in the setting of negative plain radiographs, for prompt diagnosis and treatment of pediatric SCJ injuries.
